# Selective Blockade of Interferon-α and -β Reveals Their Non-Redundant Functions in a Mouse Model of West Nile Virus Infection

**DOI:** 10.1371/journal.pone.0128636

**Published:** 2015-05-26

**Authors:** Kathleen C. F. Sheehan, Helen M. Lazear, Michael S. Diamond, Robert D. Schreiber

**Affiliations:** 1 Department of Pathology and Immunology, Washington University School of Medicine, St. Louis, Missouri, United States of America; 2 Department of Medicine, Washington University School of Medicine, St. Louis, Missouri, United States of America; 3 Department of Molecular Microbiology, Washington University School of Medicine, St. Louis, Missouri, United States of America; 4 Center for Human Immunology and Immunotherapy Programs, Washington University School of Medicine, St. Louis, Missouri, United States of America; Centro de Investigación en Medicina Aplicada (CIMA), SPAIN

## Abstract

Although type I interferons (IFNs) were first described almost 60 years ago, the ability to monitor and modulate the functional activities of the individual IFN subtypes that comprise this family has been hindered by a lack of reagents. The major type I IFNs, IFN-β and the multiple subtypes of IFN-α, are expressed widely and induce their effects on cells by interacting with a shared heterodimeric receptor (IFNAR). In the mouse, the physiologic actions of IFN-α and IFN-β have been defined using polyclonal anti-type I IFN sera, by targeting IFNAR using monoclonal antibodies or knockout mice, or using *Ifnb^-/-^* mice. However, the corresponding analysis of IFN-α has been difficult because of its polygenic nature. Herein, we describe two monoclonal antibodies (mAbs) that differentially neutralize murine IFN-β or multiple subtypes of murine IFN-α. Using these mAbs, we distinguish specific contributions of IFN-β versus IFN-α in restricting viral pathogenesis and identify IFN-α as the key mediator of the antiviral response in mice infected with West Nile virus. This study thus suggests the utility of these new reagents in dissecting the antiviral and immunomodulatory roles of IFN-β versus IFN-α in murine models of infection, immunity, and autoimmunity.

## Introduction

The type I interferons (IFNs), first identified by their ability to control viral infection [1, 2], are now known to contribute broadly to innate and adaptive immunity [3]. In mice, the type I IFN family includes IFN-β (encoded by a single gene), multiple IFN-α subtypes (14 genes and 3 pseudogenes), IFN-ζ (limitin) [4], IFN-ε [5, 6] and IFN-κ [7, 8]. The type I IFNs are encoded by single exon genes (with the exception of IFN-κ, which contains 1 intron) of similar structure, size, and conservation of protein sequence [6, 9, 10] but with divergent regulatory elements [11, 12]. Type I IFNs are induced after microbial products are sensed via pattern-recognition receptors (PRRs), which triggers activation and nuclear translocation of IRF-family transcription factors (IRF-1, -3, -5 and -7) [13–16]. While most cell types can produce IFN-β, the predominant source of IFN-α is hematopoietic cells, particularly plasmacytoid dendritic cells. [7, 17, 18].

All type I IFNs bind to the same receptor, IFNAR, a ubiquitously expressed heterodimer consisting of two subunits IFNAR1 and IFNAR2 [19–22]. Type I IFN binding to IFNAR activates the receptor associated tyrosine kinases, JAK1 and TYK2, which phosphorylate the latent transcription factors STAT1 and STAT2 to bind IRF-9. These form the ISGF3 complex, which then enters the nucleus, binds to the IFN response element in the promoters of hundreds of IFN stimulated genes (ISGs), and initiates their transcription. These ISGs promote antiviral, anti-proliferative, anti-tumor, and immunomodulatory functions [17, 23, 24]. More specifically, type I IFNs inhibit viral entry, transcription, translation, and assembly in host cells; augment host adaptive immune responses [25, 26]; and activate several key innate immune cell types including natural killer cells [27, 28], dendritic cells (DC) [29], CD8^+^ T cells [30] and B cells [31, 32]. Beyond the canonical STAT1-STAT2 signaling pathway, type I IFN-dependent activation of STAT1 and STAT3 homo- and heterodimers results in variable, context-specific, shifts in the balance of downstream signaling pathways, altering priming and induction of inflammatory responses [14, 33]. Moreover, individual type I IFN subtypes bind IFNAR with different affinities that may influence downstream gene activation [34]. Although type I IFNs trigger expression of an array of effector genes that limit viral infection [35, 36], their downstream effects also can be deleterious, leading to inflammatory immunopathology, cellular toxicity [37], cellular dysfunction [38–42] and suppression of antibacterial responses [43, 44]. Type I IFNs also have critical roles in promoting autoimmune disease [45–50] and in inducing host-protective responses to cancer [23, 51–54].

A deeper understanding of the physiologic functions of type I IFN has come from experiments using gene-targeted mice lacking *Ifnar1*, *Ifnar2* [21, 55–59], or *Ifnb* [60–62] or studies in which the various ligands or receptors are engineered to express point mutations that alter downstream signaling [63, 64]. However, distinguishing the specific *in vivo* effects of IFN-β and IFN-α has remained challenging. Better tools are needed to understand the mechanisms by which this family of cytokines functions in health and disease and how to balance protective versus harmful responses. Herein, we describe the generation and characterization of two monoclonal antibodies (mAbs), HDβ-4A7 and TIF-3C5, which selectively bind and neutralize murine IFN-β or many IFN-α subtypes, respectively. Using a mouse model of West Nile virus (WNV) infection, we demonstrate the efficacy of these neutralizing mAbs *in vivo* and identify distinct roles for IFN-β and IFN-α in controlling WNV pathogenesis. Thus, these mAbs provide a new opportunity to investigate the physiological actions of IFN-α and IFN-β *in vivo* in many biological processes.

## Materials and Methods

### Ethics Statement

These studies were carried out in strict accordance with the recommendations in the Guide for the Care and Use of Laboratory Animals of the National Institute of Health. All protocols are approved by the Washington University School of Medicine Animal Studies Committee (Animal Welfare Assurance # A3381-01). All efforts were made to minimize suffering of animals.

### Animals

Specific pathogen free C57BL/6 mice were obtained from either Jackson Laboratories (Bar Harbor, ME) or Taconic Farms (Hudson, NY). *Ifnar1* ^-/-^ [65], *Ifnb* ^*-/-*^ [60], and *Irf7* ^*-/-*^ [66] mice, all on a C57BL/6 background, were bred in the barrier animal facilities of the Washington University School of Medicine and genotyped prior to experimentation. Male and female mice from 6–18 weeks of age were used in these studies. Armenian hamsters (F1) were purchased from Cytogen Research and Development (Cambridge, MA) and housed individually with enrichment.

### Cytokines and Reagents

Recombinant murine IFN-β was purchased from BioLegend (San Diego, CA), additionally *E*. *coli*-derived IFN-β and IFN-α5 were provided by Daved Fremont (Washington University School of Medicine). Recombinant murine IFN-α1, -α4, -α11 and-α13 were provided by Anthony Coyle and Ricardo Cibotti (MedImmune, Inc., Gaithersburg, MD). Murine IFN-γ was obtained from Genentech (South San Francisco, CA) and IFN-αA/D was a gift from Hoffmann-LaRoche (Nutley, NJ). IFN-αA was purchased from PBL Assay Sciences (Piscataway, NJ). The following mAbs were produced by Leinco Technologies, Inc. (St. Louis, MO): MAR1-5A3 (neutralizing anti-murine IFNAR1) [67], GIR-208 (control murine IgG1) [68], H22 (neutralizing anti-murine IFN-γ) [69], PIP (control Armenian hamster mAb specific for bacterial glutathione-S-transferase) [70], and YTS-169 (control rat IgG). Control mouse IgG2a mAb (OKT3) was purchased from BioXCell (West Lebanon, NH). Type I IFN antibodies 7F-D3 (rat anti-IFN-β) and 4E-A1 (rat anti-IFN-α) were purchased from Abcam (Cambridge, MA). Secondary antibody reagents were purchased from Jackson ImmunoResearch (West Grove, PA).

### Production of Type I IFN specific mAbs

Mouse mAbs specific for murine IFN-β were developed using *Ifnb* ^-/-^ mice immunized by seven rounds of hydrodynamic injections of plasmid DNA encoding murine IFN-β at intervals of two-weeks or greater using methods previously described [67]. Murine *Ifnb* cDNA was cloned from a 129SvMa cDNA library derived from lipopolysaccharide and polyinosinic:polycytidylic acid (pI:C) stimulated bone marrow macrophages and subcloned into *Eco*RI and *Xba*I sites of pEF4/myc-His A vector (Invitrogen, Grand Island, NY), which adds an in-frame 3’ *myc* epitope and polyhistidine tag (5’ sense primer: TAGATTTCACCATGAACAACAGGTGGATC; 3’ antisense primer: TATCTAGAGTTTTGGAAGTTTCTGGT). The *Ifnb* sequence was excised from this vector using *Eco*RI and *Pme*I sites and ligated into pRK5 vector via *Eco*RI and *Sma*I sites and verified by Sanger sequencing. To produce IFN-β specific mAbs, immune splenocytes from genetically immunized *Ifnb* ^*-/-*^ mice were fused to the murine P3X63Ag8.653 myeloma cell line according to published procedures [67]. Supernatants from growth-positive wells were screened by ELISA for binding using plate-bound recombinant murine IFN-β compared to murine IFN-γ or IFN-α, and then for their capacity to block IFN-β effects on target cells (see below). One hybridoma cell line with neutralizing activity was identified: HDβ-4A7. This hybridoma produced a murine IgG2a mAb, which was purified by protein A affinity chromatography [67].

Antibodies reactive to IFN-α species were developed following immunization of Armenian hamsters with recombinant murine IFN-α5 alone, or with multiple IFN-α subtypes (IFN-α1, -α4, -α5, and-α11) in Freund’s adjuvant using conventional techniques [71]. Hybridomas were identified by ELISA screening where reactivity to IFN-α5, murine IFN-α1, murine IFN-α13, and human IFN-α_A/D_ but not to murine IFN-β was used as the selection criteria. TIF-3C5 and TIF-1D6 were purified by protein A affinity chromatography as described [71].

### *In vitro* IFN activity measurement

Type I IFN activity was measured using three *in vitro* functional assays: (1) induction of STAT1 phosphorylation (P-STAT1); (2) upregulation of MHC class I (MHC-I) expression; and (3) antiviral activity. P-STAT1 was measured by flow cytometry of L929 cells after incubation with mAbs and various doses of type I IFNs for 20 min at 37°C. Cells were fixed using 2% paraformaldehyde, permeabilized with 90% methanol and stained with a mAb to P-STAT1 (BD Phosflow anti-Stat1, BD Biosciences, San Jose, CA) for 1 h on ice. Enhancement of MHC-I expression was assessed by flow cytometry following treatment of a murine fibrosarcoma cell line (F510 or 1969) [72, 73] with different subtypes of IFN for 72 h in the absence or presence of mAbs as described [67]. IFN-induced antiviral effects were assessed using a cytopathic effect (CPE) assay with L929 cells and vesicular stomatitis virus (VSV, Indiana strain 5 x 10^4^ TCID_50_) as previously described [67]. In each assay, mAb was titrated as indicated in the text or Figure legends. One unit of IFN activity is defined as the volume of serum containing half-maximal stimulating activity.

### Phamacokinetics

Groups of wild-type mice were injected i.p. with 0.25 mg HDβ-4A7, TIF-3C5, or control hamster IgG (PIP) mAbs in 0.5 ml of PBS. Blood was collected from two to five mice on days 0 to 20 following mAb administration. TIF-3C5 and HDβ-4A7 levels in the serum were determined by direct ELISA using plates coated with murine IFN-α4 or IFN-β, respectively. Control Ig (PIP) was measured by indirect ELISA on anti-Armenian hamster Ig coated plates. Values were determined based on a standard curve of purified mAb diluted in normal mouse serum.

### *In vivo* pI:C stimulation

Groups of two to five mice were injected with 0.1 mg pI:C (GE Healthcare Life Science, Piscataway, NJ) and blood was collected at multiple time points as described in the text or figure legends.

### West Nile virus infection

The WNV-NY strain (3000.0259) was isolated in New York in 2000 and passaged once in C6/36 *Aedes albopictus* cells to generate a virus stock that was used in all experiments [74, 75]. WNV was diluted in Hank’s Balanced Salt Solution supplemented with 1% heat-inactivated fetal bovine serum. Groups of eight to 12 week-old age and sex matched mice were inoculated by footpad injection with 10^2^ plaque forming units (PFU) of WNV in a volume of 50 μl. Mice were anesthetized with ketamine (~1.7 mg/mouse) and xylazine (~50 μg/mouse) prior to footpad inoculation. Antibodies were delivered via intraperitoneal injection in PBS. GIR-208, PIP, or an IgG2a mAb (2H2) specific to dengue virus prM protein [76] were used as isotype controls for MAR1-5A3, TIF-3C5 and HDβ-4A7, respectively. Survival was monitored over 21 days. Mice were monitored daily for disease signs and were euthanized by controlled CO_2_ administration when they exhibited severe morbidity, including hindlimb paralysis or non-responsiveness.

### Serum analysis from WNV-infected mice

Wild-type, *Ifnar1* ^-/-^, *Ifnb* ^-/-^ or *Irf7* ^*-/-*^ mice were inoculated with WNV and serum was collected at one to six days following infection. Serum was treated at pH 3.0 for 15 min at 37°C, followed by neutralization in HEPES (final concentration 250 mM), prior to assay. This treatment inactivates WNV while preserving type I IFN activity [16].

### Statistical analysis

For serum bioassay, differences were analyzed with the Mann-Whitney test. Kaplan-Meier survival curves were analyzed by the log rank test. All data were analyzed using Prism software (GraphPad, San Diego, CA).

## Results and Discussion

### Development of mAbs against specific type I IFNs

The goal of this project was to develop mAbs that neutralized the functional activity of IFN-β or all forms of IFN-α. We employed a multi-tiered screening strategy to identify functional, subtype-specific mAbs. Having previously developed a blocking mAb specific for IFNAR1 (MAR1-5A3) using hydrodynamic genetic immunization of *Ifnar1* ^-/-^ mice [67], we used this same method to immunize *Ifnb* ^*-/-*^ mice with plasmid DNA encoding full-length murine IFN-β tagged with myc and hexahistidine. This immunization method allows for the secretion of native, glycosylated IFN-β and presentation of this ‘foreign’ antigen in an *Ifnb* ^*-/-*^ mouse with a largely intact immune system. Animals injected with plasmid DNA displayed serum titers greater than 1:30,000 as determined by ELISA using recombinant murine IFN-β. Immune sera did not detect recombinant murine IFN-α1, -α4, -α5, -α13 or IFN-γ and completely blocked IFN-β-induced STAT1 phosphorylation in L929 cells (data not shown). Fusion of immune splenocytes generated five stable hybridoma lines as assessed by selective binding to murine IFN-β by ELISA and lack of reactivity to murine IFN-γ and multiple IFN-α subtypes. Two of these IFN-β specific hybridomas secreted mAbs of the IgG2a isotype, one of which, HDβ-4A7, exhibited neutralizing activity and was selected for further characterization. A second mAb, HDβ-5F5, bound IFN-β specifically but did not block its function. The three other IFN-β-specific hybridomas secreted IgM and were not analyzed further.

We next sought to produce a “pan-IFN-α” mAb that would neutralize all 14 murine IFN-α subtypes (75–99% amino acid identity) [6, 9, 10] but not IFN-β (~30% identity) [77]. We could not use the same approach that generated the IFN-β mAbs because mice lacking the multi-gene IFN-α complex have not been reported. Instead, we immunized Armenian hamsters with several different combinations of murine IFN-α proteins including injection with a single IFN-α subtype (IFN-α5) or with multiple IFN-α subtypes (IFN-α1, -α4, -α5, and-α11) either mixed together or following sequential immunizations. Each of the immunization strategies generated polyclonal neutralizing antisera with similar titers against IFN-α5. Immune serum derived from the hamster immunized repeatedly with purified recombinant murine IFN-α5 displayed the highest titer (>1:25,000), as assessed with an IFN-α5 ELISA, and showed no binding to IFN-β. This immune serum also blocked IFN-α5-induced STAT1 phosphorylation in L929 fibroblasts (data not shown). Following fusion, many hybridomas were identified that showed ELISA reactivity with multiple subtypes of recombinant murine IFN-α. However, only mAb derived from the TIF-3C5 hybridoma neutralized all of the IFN-α subtypes tested. In comparison, mAbs derived from other hybridomas bound or blocked different subsets of the IFN-αs used (S1 Table).

### *In vitro* neutralization of type I IFN activity

**Fig 1:**
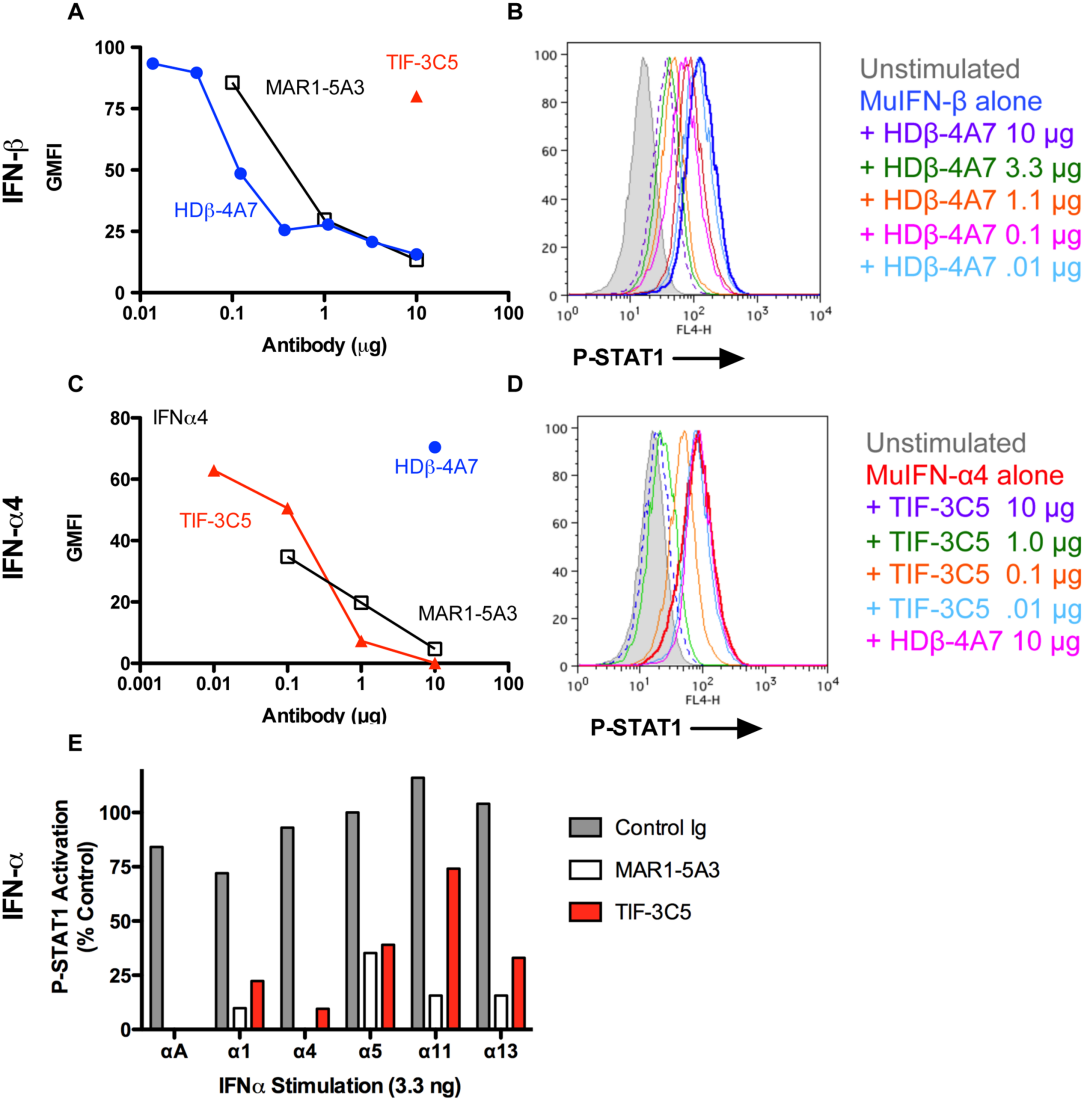
Blockade of type I IFN activation of P-STAT1 *in vitro*. Titrations of HDβ-4A7 and TIF-3C5 (0.01–10 μg) or MAR1-5A3 (10 μg) were preincubated for 60 min with 3 ng of IFN-β (**A, B**) or 3.3 ng of IFN-α4 (**C, D**) and then incubated with L929 cells for 20 min, followed by staining for P-STAT1 and processing by flow cytometry. **E**. Individual IFN-α subtypes (3.3 ng) were preincubated with 10 μg of mAb and then incubated with L929 cells for 20 min, followed by staining for P-STAT1 and flow cytometric analysis. Data are representative of three or more independent experiments.

Three biological assays were used to identify mAbs that neutralized specific type I IFNs: (1) induction of STAT1 phosphorylation; (2) upregulation of cell surface MHC-I expression; and (3) inhibition of CPE caused by VSV infection. We initially measured the ability of HDβ-4A7 and TIF-3C5 to block IFN-β- or IFN-α-induced STAT1 phosphorylation, as this represents a rapid response following ligand binding to IFNAR1/IFNAR2 that occurs within minutes of receptor engagement. HDβ-4A7 inhibited STAT1 phosphorylation in a dose-dependent manner, comparable to that achieved using an IFNAR1 blocking mAb, MAR1-5A3 (Fig 1A and 1B). A second IFN-β-specific IgG2a mAb, HDβ-5F5, as well as several IFN-α-specific mAbs, failed to block STAT1 phosphorylation induced by IFN-β (data not shown). To examine inhibition of IFN-α activity *in vitro*, we tested STAT1 phosphorylation in response to IFN-α4 stimulation. TIF-3C5 inhibited IFN-α4-mediated phosphorylation in a dose-dependent manner, similar to that achieved using anti-IFNAR1 mAb, whereas incubation of IFN-α4 with the anti-IFN-β mAb HDβ-4A7 did not alter phospho-STAT1 levels (Fig 1C and 1D). TIF-3C5 also blocked induction of STAT1 phosphorylation in response to recombinant IFN-αA, IFN-α1, IFN-α5, IFN-α11 (partial blockade), and IFN-α13 (Fig 1E), with no inhibition of IFN-β (data not shown). Thus, TIF-3C5 exhibited neutralizing activity for all six of the recombinant IFN-α subtypes tested.

**Fig 2:**
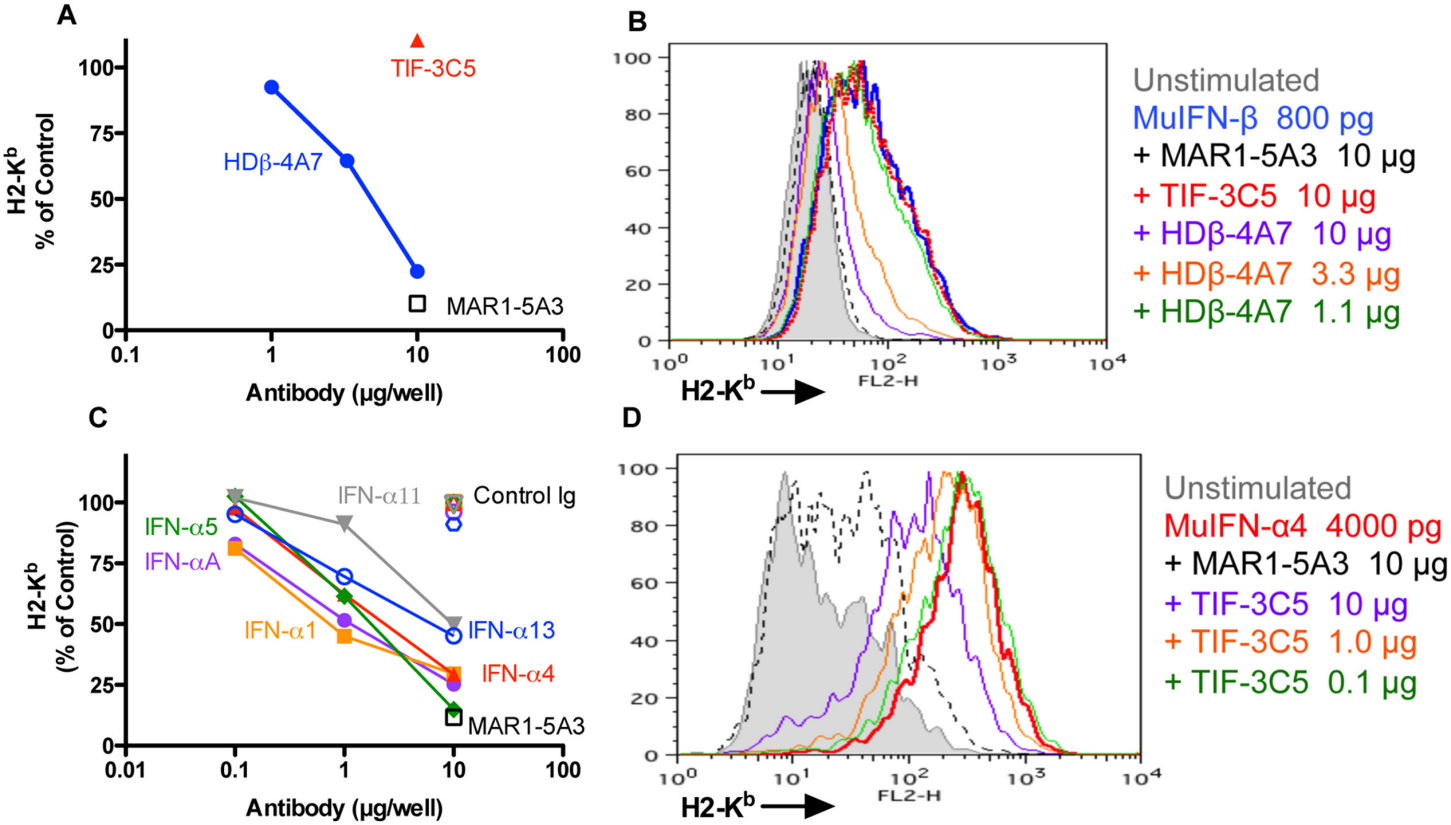
Inhibition of type I IFN MHC I upregulation. Type I IFNs (IFN-β, 800 pg; IFN-α1, 40,000 pg; IFN-α4, 4000 pg; IFN-α5, 400 pg; IFN-α11, 1000 pg; or IFN-α13, 1000 pg) were preincubated with 1 to 10 μg of HDβ-4A7 (**A, B**) or 0.1 to 10 μg of TIF-3C5 (**C, D**), MAR1-5A3 (10 μg) or control hamster IgG (10 μg) for 30 min then added to fibrosarcoma cells for 72 h and stained for H2-K^b^ class I MHC antigens by flow cytometry. Plots and histograms are representative of three or more independent assays using either F510 (**A, B**) or 1969 (**C, D**) fibrosarcoma cells.

We next examined type I IFN induction of cell surface expression of MHC-I. We used the mouse sarcoma lines F510 or 1969 that constitutively express low levels of MHC class I, but upregulate cell surface expression of H-2K^b^ and H-2D^b^ following IFN treatment. HDβ-4A7 efficiently blocked IFN-β-mediated H2-K^b^ expression whereas TIF-3C5 had no effect (Fig 2A and 2B). In parallel experiments, TIF-3C5 inhibited MHC-I upregulation in a dose dependent manner upon stimulation with IFN-α4 (Fig 2C and 2D) or additional IFN-α subtypes (IFN-αA, -α1, -α5, -α11 and-α13), consistent with TIF-3C5 reactivity against all species of IFN-α tested. HDβ-4A7 and TIF-3C5 blocked IFN-β and IFN-α activity, respectively, in a manner that was similar to IFNAR blockade by MAR1-5A3.

**Fig 3:**
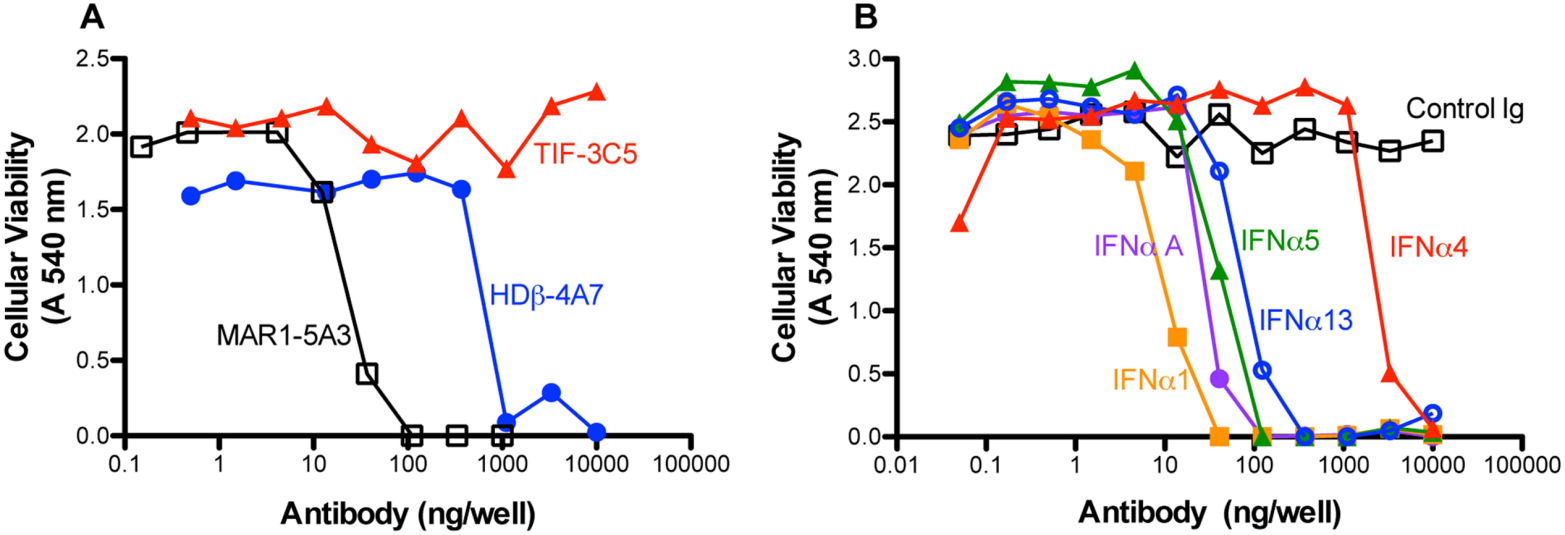
Neutralization of type I IFN induced antiviral activity *in vitro*. Ten units IFN-β (**A**) or IFN-α (**B**) were preincubated for 1 h with indicated mAbs and then added to L929 cells overnight. Ten units/well type I IFN activity used in these assays are as follows: IFN-β, 16 pg; IFN-αA, 22 pg; IFN-α1, 15 pg; IFN-α4, 70 pg; IFN-α5, 20 pg; IFN-α13, 13 pg. IFN-treated cells subsequently were infected with VSV for 48 h and cell viability determined by crystal violet staining and optical density measurements. Results shown are representative of three or more independent experiments.

We also assessed the ability of HDβ-4A7 and TIF-3C5 to neutralize type I IFN antiviral activity using a cytopathic effect bioassay. HDβ-4A7 neutralized 10 U (16 pg) of IFN-β antiviral activity with a 50% inhibitory dose (ID_50_) of 600 ng/well, compared to 30 ng/well for the IFNAR1-binding mAb, MAR1-5A3 (Fig 3A). As expected, TIF-3C5 did not inhibit IFN-β antiviral activity. Although TIF-3C5 completely blocked the antiviral activity of each IFN-α subtypes tested, it did so with differing efficiencies (Fig 3B). IFN-α1 was blocked at low concentrations of mAb (ID_50_ = 9 ng/well) whereas more TIF-3C5 was needed to inhibit IFN-α4 (ID_50_ = 2000 ng/well). It is unclear in these studies if this spectrum of activity reflects the unique binding affinity of TIF-3C5 for each IFN-α subtype, the specific activity of each of the IFN-α subtype used, or if particular IFN-α subtypes are more effective at triggering specific biological functions. Hence, we have identified two mAbs, HDβ-4A7 and TIF-3C5, capable of selectively neutralizing the *in vitro* activities of recombinant IFN-β and IFN-α, respectively.

**Fig 4:**
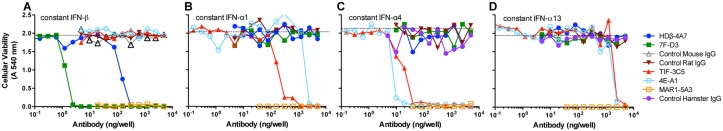
*In vitro* neutralizing activity of Type I IFN mAbs. Approximately 20 units of recominant IFN-β (**A**) or IFN-α1 (**B**), IFN-α4 (**C**) or IFN-α13 (**D**) were preincubated for 1 h with indicated mAbs and then added to L929 cells overnight. Constant levels of Type I IFN used in these assays are as follows: IFN-β, 4.4 ng; IFN-α1, 0.15 ng; IFN-α4, 0.05 ng; IFN-α13, 34 ng. IFN-treated cells subsequently were infected with VSV for 48 h and cell viability determined by crystal violet staining and optical density measurements. Results shown are representative of two independent experiments.

We compared the *in vitro* neutralizing activity of HDβ-4A7 and TIF-3C5 to previously reported rat mAbs targeting IFN-β and IFN-α (7F-D3 and 4E-A1, respectively) [78]. HDβ-4A7 exhibited less potent neutralizing activity than 7F-D3, and the relative potencies of TIF-3C5 and 4E-A1 depended on the subtype of IFN-α tested (Fig 4). While 7F-D3 and 4E-A1 exhibit neutralizing activity against murine IFN-β and IFN-α, anti-globin responses can effect mAb pharmacokinetics, making these existing rat IgG reagents problematic for repeated *in vivo* administration [79]. The new mAbs reported in this work are more amenable to *in vivo* use, as HDβ-4A7 is of murine origin and TIF-3C5 is derived from Armenian hamster, a species whose IgG is not immunogenic in mice [70,78]

### *In vivo* production of type I IFN

**Fig 5:**
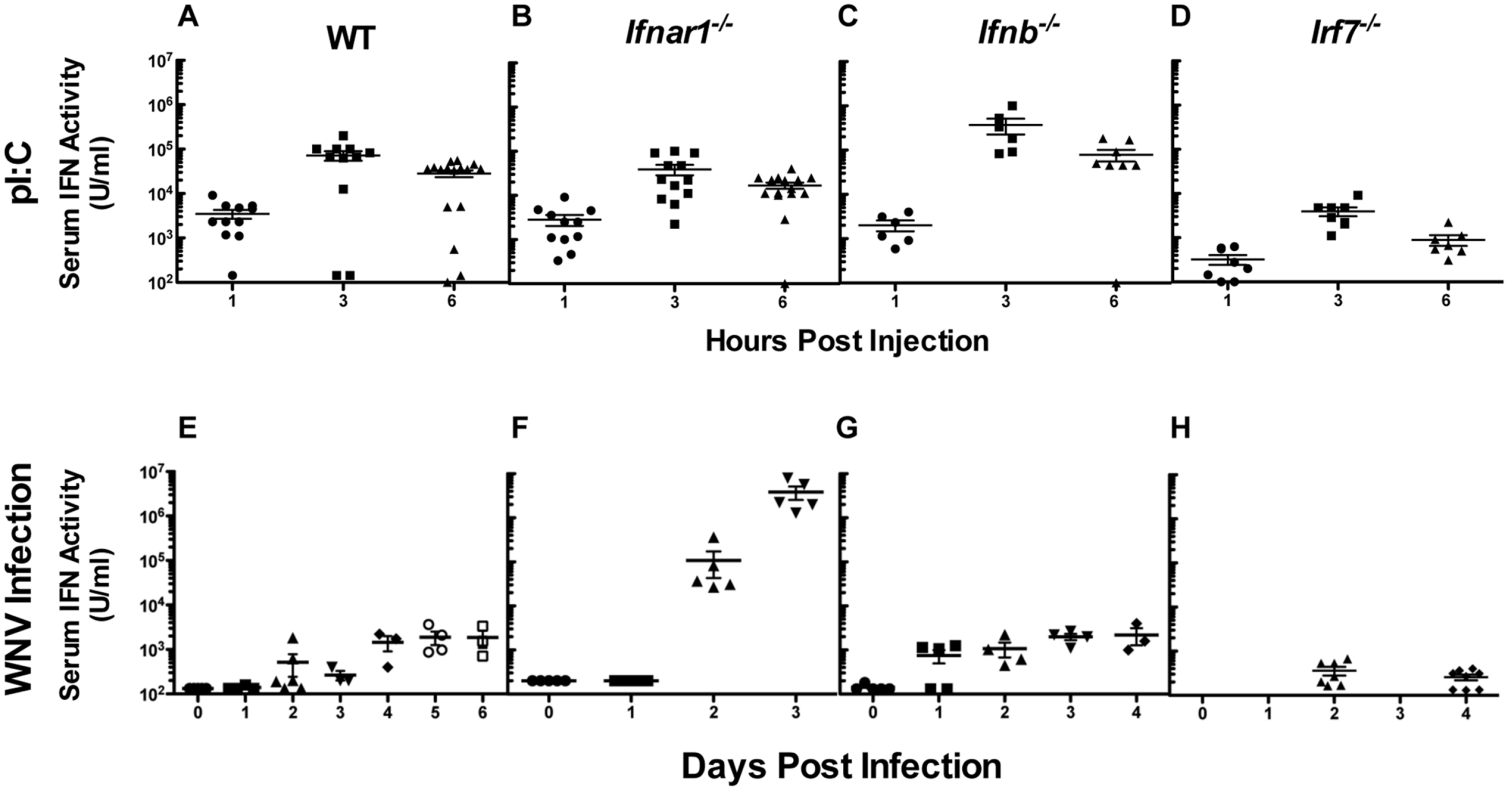
Kinetics of native type I IFN production *in vivo*. Wild-type (**A, E**), *Ifnar1* ^-/-^ (**B, F**), *Ifnb* ^-/-^ (**C, G**) or *Irf7* ^-/-^ (**D, H**) mice were treated with 100 μg of pI:C (**A-D**) or infected with 10^2^ PFU of WNV (**E-H**) and serum was assayed on the indicated times for IFN activity by VSV CPE bioassay. Samples from three independent sets of pI:C treated mice (3 to 5 per group) were collected at 1, 3 and 6 h post injection. Serum from groups of 3 to 8 mice of each genotype was collected from naïve and WNV infected mice at days 1 to 6 post infection. Each genotype was sampled from at least two (*Ifnar1* ^-/-^ and *Irf7* ^-/-^) or more (wild-type and *Ifnb* ^-/-^) independent infections. IFN levels in all samples were determined at least three times. As shown herein, symbols represent samples derived from individual animals at the times indicated and assayed at the same time.

We next tested the ability of HDβ-4A7 and TIF-3C5 to neutralize type I IFNs generated *in vivo* in response to inflammatory stimuli or infection. *Ifnar1* ^-/-^, *Ifnb* ^-/-^ or *Irf7* ^-/-^ mice were used to generate distinct mixtures of IFN compared to those generated using wild-type animals. *Ifnar1* ^-/-^ mice produce high levels of circulating IFNs as they lack a receptor to bind and internalize type I IFNs. *Ifnb* ^-/-^ mice do not produce IFN-β, but generate IFN-α and have normal responses to type I IFN [60, 61]. *Irf7* ^-/-^ mice produce IFN-β, however IFN-α production is largely ablated [66, 80]. In the first set of experiments, mice were treated with the TLR3/MDA5 agonist pI:C to induce IFN production, and serum was collected as a source of naturally produced IFN. Antiviral activity in the serum was quantitated using the VSV CPE bioassay (Fig 5A–5D). Wild-type mice injected with pI:C produced peak type I IFN activity at 3 h post injection, with levels declining rapidly by 12 h (data not shown). The kinetics of production were identical for each of the gene-targeted strains examined with peak levels similar to those described using pI:C treated wild-type or reporter mice [81, 82]. *Ifnb* ^-/-^ mice produced slightly elevated peak levels of type I IFN, whereas *Irf7* ^-/-^ animals generated lower levels overall.

We infected mice with WNV, an encephalitic flavivirus, and collected sera one to six days after infection. Again, we quantitated serum IFN activity using the VSV CPE bioassay (Fig 5E–5H). Serum levels of IFN-α and IFN-β rose rapidly in *Ifnar1* ^-/-^ mice, which sustain extremely high levels of viral replication and viral nucleic acids, further driving IFN production [16]. As previously observed, serum antiviral activity in *Ifnb* ^-/-^ mice was similar to that of wild-type mice, implying that a substantial component of the antiviral activity in wild-type serum comes from IFN-α [61]. As expected, only minimal antiviral activity was detected in the serum from *Irf7* ^-/-^ mice [80].

### Neutralization of *in vivo* generated IFN-β and IFN-α

**Fig 6:**
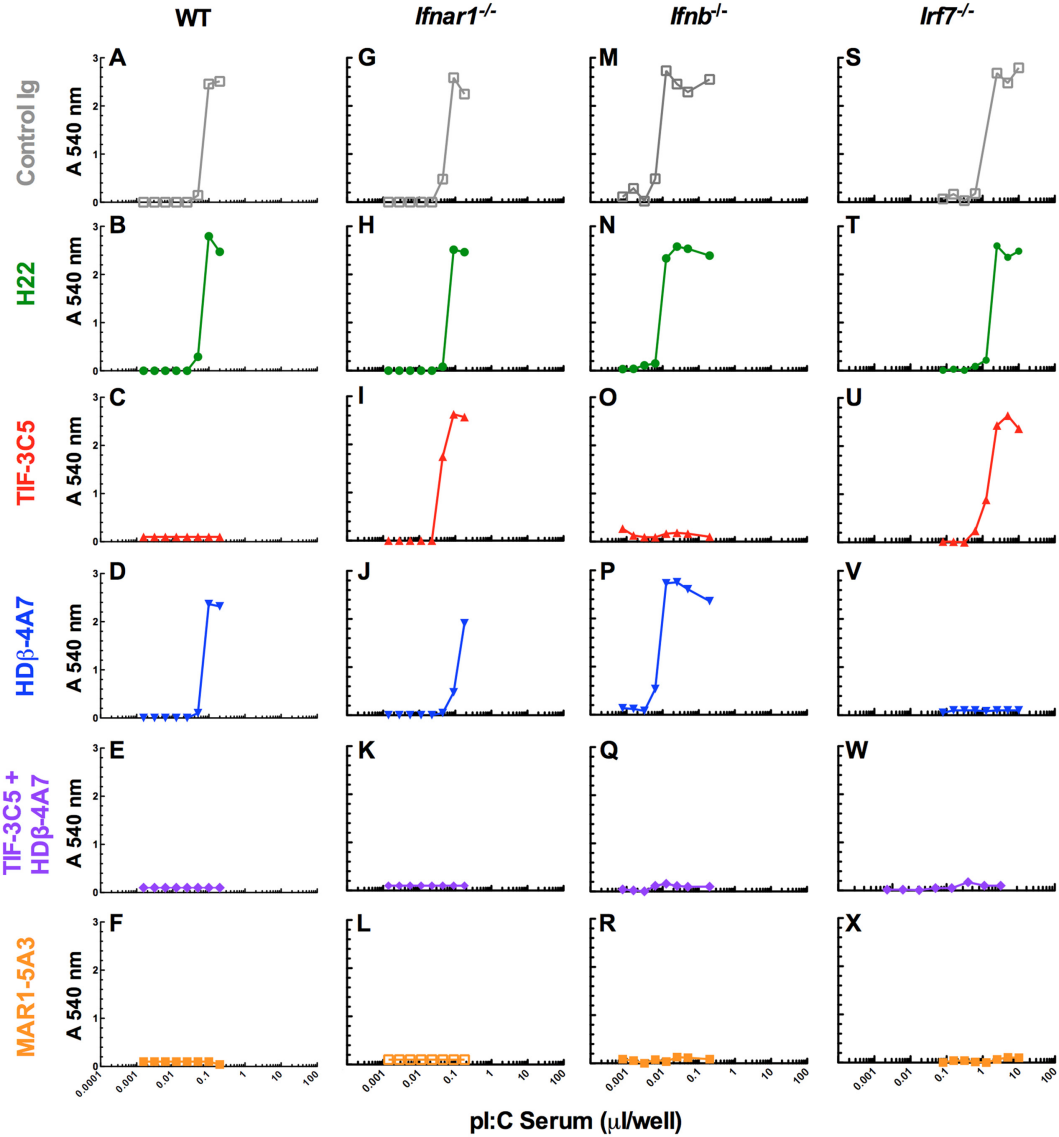
Detection of IFN-β and IFN-α in serum following pI:C treatment. Serum was collected from wild-type (**A-F**), *Ifnar1* ^-/-^ (**G-L**), *Ifnb* ^-/-^ (**M-R**) or *Irf7* ^-/-^ (**S-X**) mice injected with 100 μg of pI:C at 3 to 6 hrs. Volumes of serum containing approximately 5–10 units of IFN activity (as determined in Fig 4) were incubated with 10 μg of mAb for 1 h and then titrations were added to L929 cells overnight. IFN-treated cells were infected with VSV and cellular viability assessed after 48 h using crystal violet staining and optical density measurements. Each panel is representative of six independent samples.

**Fig 7:**
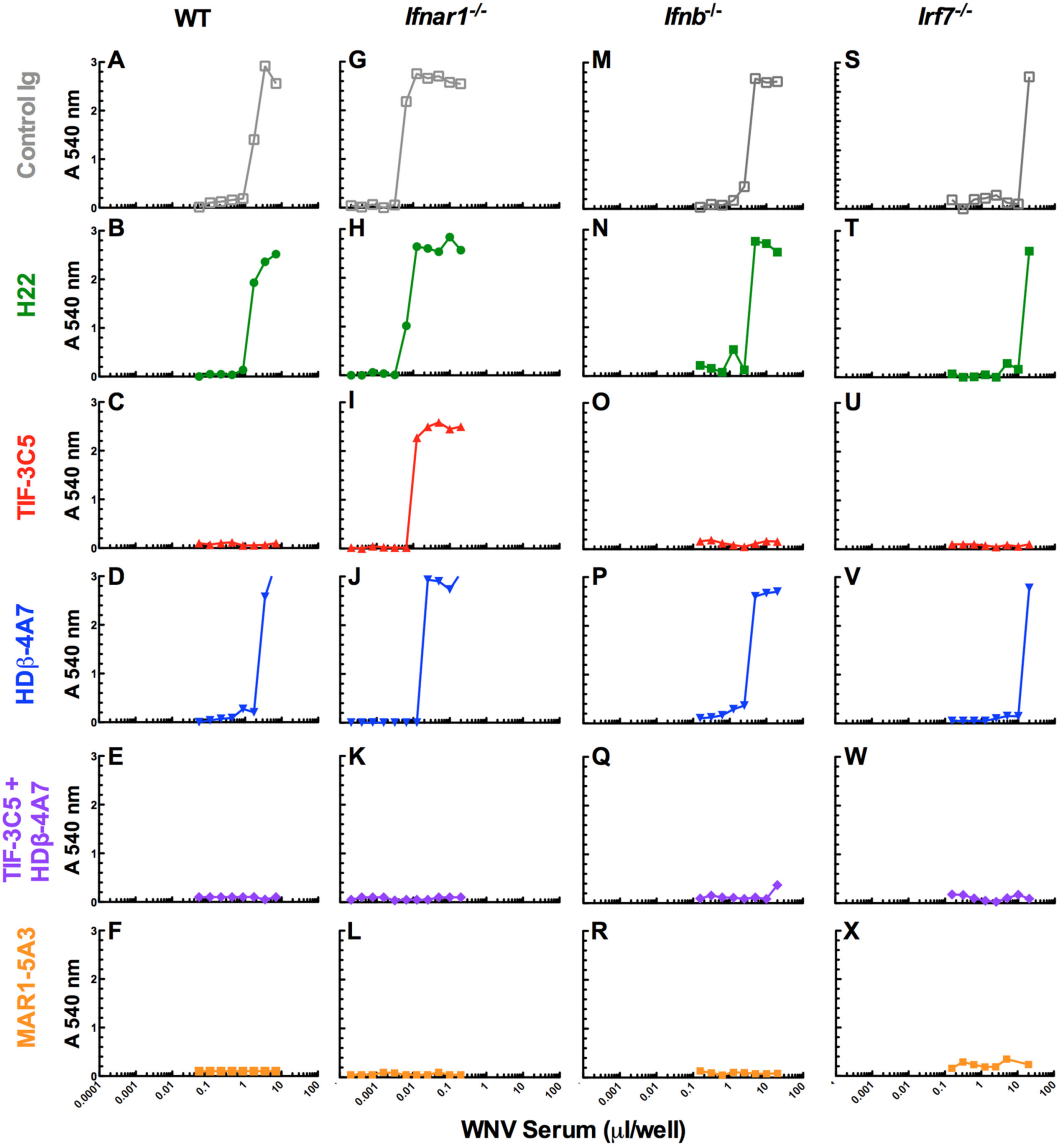
Detection of IFN-β and IFN-α in serum following WNV infection. Serum was collected from wild-type (**A-F**), *Ifnar1* ^-/-^ (**G-L**), *Ifnb* ^-/-^ (**M-R**) or *Irf7* ^-/-^ (**S-X**) mice infected with 10^2^ PFU of WNV at day 3 (wild-type and *Ifnar1* ^*-/-*^) or day 4 (*Ifnb* ^*-/-*^ *and Irf7* ^*-/-*^). Volumes of serum containing approximately 5–10 units of IFN activity (as determined in Fig 4) were incubated with 10 μg of mAb for 1 h and then titrations were added to L929 cells overnight. IFN-treated cells were infected with VSV and cellular viability assessed after 48 h using crystal violet staining and optical density measurements. Each panel is representative of six independent samples.

We used our neutralizing mAbs to characterize the composition of natural IFN produced by wild-type, *Ifnar1* ^-/-^, *Ifnb* ^-/-^ or *Irf*7^-/-^ mice in response to pI:C treatment (Fig 6) or WNV infection (Fig 7). IFN-containing sera were incubated with control or neutralizing mAbs and the antiviral activity measured by the VSV CPE bioassay. All of the antiviral activity present in the serum samples was attributable to type I IFN because it was abrogated by addition of the IFNAR1-blocking mAb MAR1-5A3 (Figs 6 and 7, panels F, L, R and X). Consistent with this observation, treatment with an IFN-γ neutralizing mAb, H22, did not affect the antiviral activity (Figs 6 and 7, panels B, H, N and T), which was identical to cultures treated with buffer or control IgG. In all cases, the combination of both HDβ-4A7 and TIF-3C5 ablated all antiviral activity in the bioassay (Figs 6 and 7, panels E, K, Q and W), similar to that seen with MAR1-5A3, revealing that other type I IFNs (e.g., IFN-κ or IFN-ζ) or type III IFNs (IFN-λ) did not contribute to antiviral activity under these conditions. In sera from wild-type mice stimulated with pI:C for 6 h, the type I IFN activity was blocked completely by TIF-3C5 with no effect of neutralization with HDβ-4A7, indicating that IFN-α was the only type I IFN produced at this time point (Fig 6C and 6D). Identical results were obtained using serum samples obtained at 3 h post injection of pI:C (data not shown). Analogously, in serum obtained 3 days after WNV infection of wild-type mice, TIF-3C5 fully blocked antiviral activity, with HDβ-4A7 having virtually no impact, indicating that IFN-α is the dominant type I IFN in serum following WNV infection at that time point (Fig 7C and 7D). Neither TIF-3C5 nor HDβ-4A7 alone could completely neutralize the antiviral activity present in serum from *Ifnar1* ^-/-^ mice following either pI:C treatment or WNV challenge, demonstrating that both IFN-β and IFN-α are produced in these immunodeficient mice in response to these stimuli although IFN-α predominates (Figs 6 and 7, panels I and J). As expected, HDβ-4A7 had no impact on the antiviral activity found in serum from *Ifnb* ^-/-^ mice and all of the IFN activity in these samples was neutralized by TIF-3C5 (Figs 6 and 7, panels O and P). With serum from pI:C-treated *Irf7* ^-/-^ mice, HDβ-4A7 neutralized antiviral activity whereas TIF-3C5 had no effect (Fig 6V and 6U). In contrast, TIF-3C5 neutralized all of the type I IFN activity from WNV-infected *Irf7* ^-/-^ mice, with no neutralization by HDβ-4A7 (Fig 7U and 7V). Thus, IFN-α was generated in *Irf7* ^-/-^ mice in response to WNV infection but not after pI:C treatment. This IFN-α activity may be due to the IFN-α4 subtype, which is an unusual IFN-α that can be induced directly in response to pattern recognition receptor signaling and does not require IFN-β production or IRF-7-dependent transcriptional activity for its expression [83]. Although *Irf7* ^-/-^ mice would be expected to induce some IFN-β in response to WNV infection, this low level of IFN-β may be induced at early time points and cleared from the circulation by IFNAR1 or fall below the sensitivity of this assay. Collectively, these data establish that HDβ-4A7 and TIF-3C5 can bind and neutralize naturally-produced IFN-β and IFN-α, respectively, and provide a sensitive tool to interrogate the appearance and quantity of either IFN in complex biological samples such as serum.

### Type I IFN mAbs enhance lethality of WNV infection

Having identified mAbs that selectively neutralize IFN-β and IFN-α *in vitro*, we assessed whether they could neutralize IFN activity *in vivo*. Pharmacokinetic analysis revealed circulatory half-lives of 14 days for HDβ-4A7 and 3.5 days for TIF-3C5 as determined by ELISA using IFN-β and IFN-α5, respectively, and 4 days for PIP control Armenian hamster IgG (data not shown). To evaluate the ability of HDβ-4A7 and TIF-3C5 to neutralize IFNs *in vivo*, we used a well-characterized model of WNV infection in which type I IFNs control viral replication and pathogenesis [61, 84, 85].

**Fig 8:**
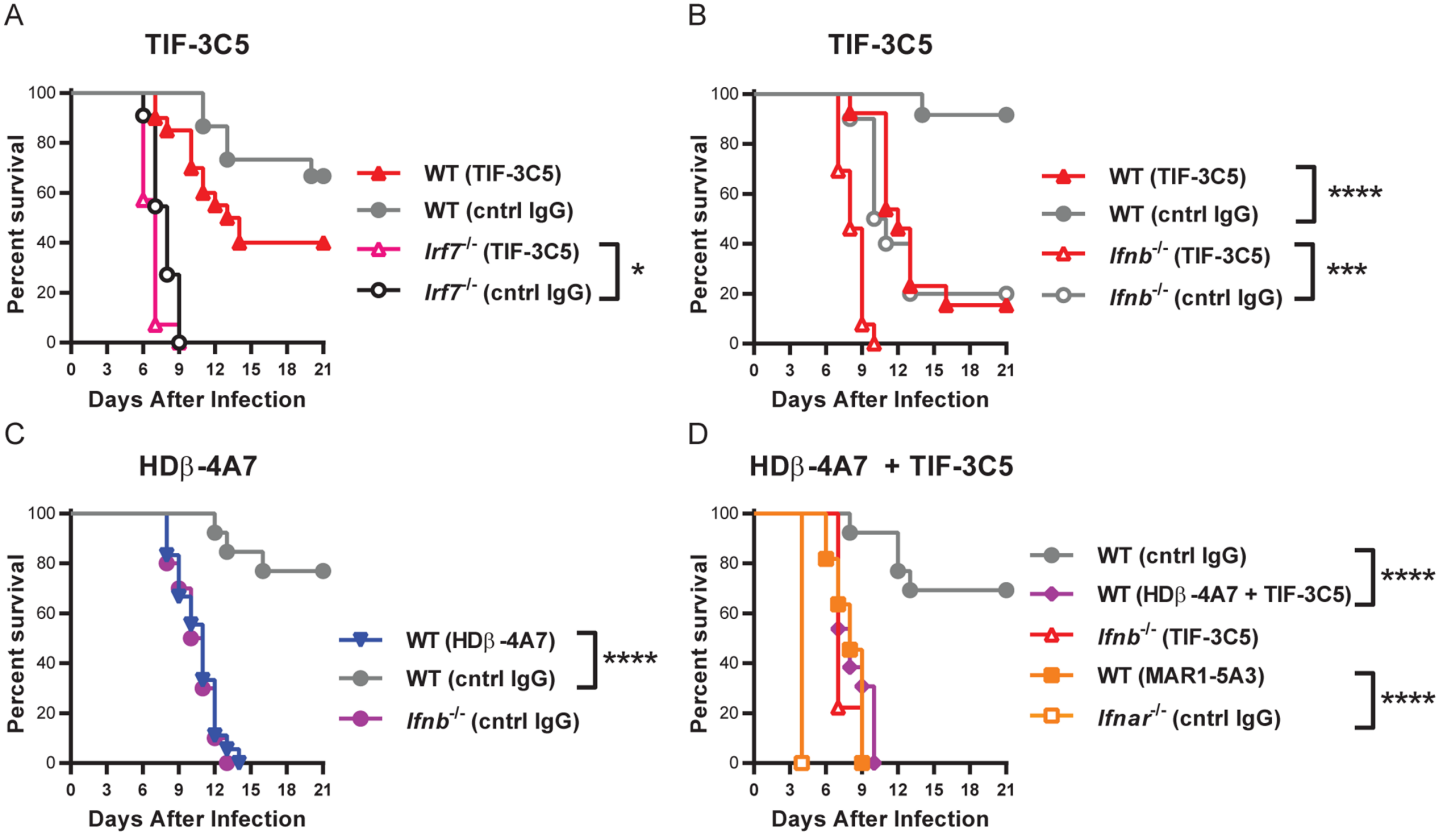
Type I IFN blocking mAbs enhance lethality of WNV infection. Wild-type or *Irf7* ^*-/-*^ mice were administered 500 μg of TIF-3C5 or isotype control mAb by intraperitoneal injection one day prior, and two days following subcutaneous infection with 10^2^ PFU of WNV. **B.** Wild-type or *Ifnb* ^-/-^ mice received 250 μg of TIF-3C5 or isotype control mAb one day prior, one day following, and three days following WNV infection. **C.** Wild-type or *Ifnb* ^-/-^ mice received 250 μg of HDβ-4A7 or isotype control antibody one day prior and two days following WNV infection. **D.** Wild-type or *Ifnb* ^-/-^ mice received 250 μg of HDβ-4A7 antibody or isotype control one day prior and three days following WNV infection, as well as 500 μg of TIF-3C5 antibody (or control) one day prior, one day following, and three days following infection. Wild-type and *Ifnar1* ^-/-^ mice were given 500 μg of MAR1-5A3 or isotype control mAb one day prior, one day following, and three days following infection. Survival was monitored for 21 days. Data represent 9 to 20 mice per group, from 2 or more independent experiments. *, *P* < 0.05; ***, *P* < 0.001; ****, *P* < 0.0001 (log-rank test).

*In vivo* mAb efficacy was assessed by monitoring survival following WNV infection. We observed increased lethality in wild-type mice treated with 500 μg of TIF-3C5 one day prior and two days following WNV infection, compared to mice receiving isotype control IgG (Fig 8A), but this difference did not achieve statistical significance (*P* > 0.05). We tried an alternate dosing regimen and found that three administrations of TIF-3C5 (250 μg one day prior, one day following, and three days following WNV infection) produced a greater and statistically significant increase in lethality compared to two doses of 500 μg (Fig 8B). TIF-3C5 treatment enhanced lethality compared to isotype control mAb in both wild-type and *Ifnb* ^-/-^ mice, indicating a key antiviral role for IFN-α in the presence or absence of IFN-β [86]. Wild-type mice treated with 250μg of HDβ-4A7 one day prior and two days following WNV infection succumbed with kinetics identical to *Ifnb* ^-/-^ mice, indicating that HDβ-4A7 completely neutralizes IFN-β activity *in vivo* (Fig 8C). Combined administration of both HDβ-4A7 and TIF-3C5 in wild-type mice, or TIF-3C5 treatment in *Ifnb* ^-/-^ mice, phenocopied treatment with MAR1-5A3 (Fig 8D). Since IFN-β and IFN-α fully accounted for the type I IFN response observed, these data suggest that other type I IFN subtypes do not contribute significantly to the antiviral response to WNV in this system. TIF-3C5 treatment produced a small increase lethality in *Irf7* ^*-/-*^ mice (Fig 8A), consistent with the ability of this mAb to neutralize antiviral activity in serum from WNV-infected *Irf7* ^-/-^ mice (Fig 7U). We conclude that while some residual IFN-α is induced in the absence of IRF-7, it is insufficient to support an antiviral response, perhaps because IRF-7 is necessary for the expression of antiviral effector molecules in response to IFN-α signaling. We presume that the IRF-7-independent IFN-α produced is IFN-α4. Together, our observations provide new insights into the specific roles of IFN-β and IFN-α in the response to WNV infection. Specifically, we detected very little IFN-β in serum of WNV infected mice (days 2 to 6 after infection) and found that the type I IFN response was dominated by IFN-α; it is likely that small quantities of IFN-β are sufficient to initiate positive feedback amplification driving IFN-α production. Since TIF-3C5 neutralizes all murine IFN-α subtypes tested, the present studies do not distinguish unique functions for different IFN-α subtypes *in vivo*; neutralizing mAbs targeting individual or defined sets of IFN-α subtypes will be needed to determine their specific properties. Although our observations define the specific contributions of IFN-α versus IFN-β in serum, other IFN subtypes may act in a localized manner in particular cells or tissues. Furthermore, the relative contributions of IFN subtypes to the antiviral response for different infections may depend on the tropism and evasion strategies of the virus studied.

In summary, we generated two new mAbs, HDβ-4A7 and TIF-3C5, that are highly specific for murine IFN-β and most, if not all, IFN-α subtypes, respectively, and can block type I IFN activity *in vitro* and *in vivo*. We used these novel reagents to distinguish specific contributions of IFN-β and IFN-α to the antiviral response to WNV infection. Our observations reveal a complex interplay between IFN production and host survival from infection. These unique reagents provided us with an ability to monitor and/or selectively ablate the functional activity of individual type I IFN subtypes in complex models of immune and inflammatory stimulation. Unlike studies in gene-targeted animals, these reagents will permit modulation of IFN activity at particular time points relative to infectious or inflammatory stimuli in the context of an immune system that has developed in a normal IFN milieu. These mAbs will be valuable tools to interrogate the specific functional roles of IFN-β and IFN-α in inflammatory, infectious, or autoimmune models of disease.

## Supporting Information

S1 ARRIVE Checklist (PDF)

S1 Table ELISA Binding Specificity of Type I IFN Antibodies. Binding of mAbs to adsorbed recombinant IFNs was measured by ELISA.
(DOCX)
