# Supplementary material for: Selective Blockade of Interferon-α and -β Reveals Their Non-Redundant Functions in a Mouse Model of West Nile Virus Infection
Source: PLoS One. 2015 May 26;10(5):e0128636. doi: 10.1371/journal.pone.0128636 (PMC4444312; doi:10.1371/journal.pone.0128636)
Supplement: S1 Table — Binding of mAbs to adsorbed recombinant IFNs was measured by ELISA. (DOCX) [file pone.0128636.s002.docx]

**Supporting Information**

**Table S1. ELISA Binding Specificity of Type I IFN Antibodies^a^**

| **mAb** | **IFN-β** | **IFN-αA** | **IFN-α1** | **IFN-α4** | **IFN-α5** | **IFN-α11** | **IFN-α13** | **HuIFN-**  **αA/D** | **IFN-γ** |
| --- | --- | --- | --- | --- | --- | --- | --- | --- | --- |
| HDβ-4A7 | **+++** | **-** | **-** | **-** | **-** | nd | **-** | nd | **-** |
| HDβ-5F5^b^ | **+++** | **-** | **-** | **-** | **-** | nd | **-** | nd | **-** |
| TIF-3C5 | **-** | **+++** | **+++** | **+++** | **+++** | **+++** | **+++** | **-** | **-** |
| TIF-1D6^b^ | **-** | nd | **+++** | **+** | **+++** | nd | **+++** | **++** | **-** |
| IFI-2A11 | **-** | **+/-** | **+++** | **-** | **+++** | **-** | **+++** | nd | **-** |

^a^ Binding of mAbs to adsorbed IFNs was measured by ELISA. mAbs were designated as high-binding (+++), intermediate binding (++), low binding (+), or non-binding (-).

nd, not determined.

^b^  non-neutralizing
